# Source apportionment of fine atmospheric particles in Bloemfontein, South Africa, using positive matrix factorization

**DOI:** 10.1007/s10661-023-12293-4

**Published:** 2024-01-23

**Authors:** Deidré van der Westhuizen, Chantelle Howlett-Downing, Peter Molnár, Johan Boman, Janine Wichmann, Karel G. von Eschwege

**Affiliations:** 1 Department of Chemistry, University of the Free State, PO Box 339, Bloemfontein, 9300 South Africa; 2 School of Health Systems and Public Health, Faculty of Health Sciences, University of Pretoria, Pretoria, South Africa; 3 Department of Occupational and Environmental Medicine, Institute of Medicine, Sahlgrenska Academy, University of Gothenburg, Gothenburg, Sweden; 4 Department of Chemistry and Molecular Biology, Atmospheric Science Division, University of Gothenburg, Gothenburg, Sweden

**Keywords:** Air pollution, PM_2.5_, Trace elements, Black carbon, Organic carbon, Source apportionment

## Abstract

Air pollution is of major health and environmental concern globally and in South Africa. Studies on the sources of PM_2.5_ air pollution in low- and middle-income countries such as South Africa are limited. This study aimed to identify local and distant sources of PM_2.5_ pollution in Bloemfontein. PM_2.5_ samples were collected from June 16, 2020 to August 18, 2021. Trace element concentrations were determined by EDXRF spectroscopy. By use of the US EPA PMF 5.0 program, local sources were determined to be combustion/wood burning (49%), industry (22%), soil dust (10%), base metal/pyrometallurgical and traffic (9.6%) and water treatment/industry (9.4%). The HYSPLIT program was applied to determine distant PM_2.5_ source areas and the following clusters were identified: Mpumalanga province (52%), Northern Cape province (35%), Indian Ocean (8%) and Atlantic Ocean (6%). The majority of the air was found to come from the Mpumalanga province in the north-east, while the majority of local sources are ascribed to combustion/wood burning. Results from this study can be used to develop an Air Quality Management Plan for Bloemfontein.

**Supplementary Information:**

The online version contains supplementary material available at 10.1007/s10661-023-12293-4.

## Introduction

High level emissions of various air pollutants in South Africa (SA) are of major concern, resulting not only in outdoor but also indoor air pollution (Adeyemi et al., 2021). Pollution from various sources has an effect on air quality. These sources pose threats to the environment and affect the ecosystem’s ability to sustain and preserve life, thus linking to several of the global sustainable development goals (SDG) (Sustainable-development-goals, 2023). SDGs such as sustainable cities and communities, industry, innovation and infrastructure, clean water and sanitation, and finally responsible consumption and production contribute to air pollution through the processes required to achieve these goals. In addition, air pollution impacts the following SDGs: climate action, affordable clean energy and health and well-being. To achieve clean energy goals, SA should utilize its rich resource of solar power instead of fossil fuel (coal) power generation, which will result in reducing the causes of air pollution and thus the effects of global warming. PM_2.5_ pollution has the ability to affect human health and well-being, due to the inhalability of the particles, causing diseases such as cancer, cardiovascular disease, infertility and asthma (Sustainable-development-goals, 2023). Because of the negative impact it poses to human health, ambient and household particulate matter smaller or equal to 2.5 µm in diameter (PM_2.5_) received much attention during the last decade. Relationships between health problems and particulate matter (PM) were reported in several published epidemiology studies (Rupp, 2009; Brunekreef & Holgate, 2002; Kappos et al., 2004; Lokotola et al., 2020; Schwartz, 2004).

Data on source apportionment using particulate matter in the South African region are limited (Karagulian et al., 2015). Studies in this region have found high levels of industrial air pollution due to the large metallurgical and mining sectors (Tiitta et al., 2014; Van Zyl et al., 2014). South Africa has a wide variety of climatic types and therefore sources vary depending on the location of the sampling sites. For example, Cape Town is influenced by sources such as sea salt due to its proximity to a coastal area; Pretoria is influenced by mineral dust, industrial and anthropic activities due to its urban/industrial inland location; and Bloemfontein is influenced by biomass burning due to its suburban and rural location with high agricultural activities (Kumar et al., 2014). Table 1 shows the different sources found in previous studies according to their location (Mathuthu et al., 2019).

**Table 1:** Source categories found in previous studies

Site	Source categories
Urban	Anthropogenic (e.g. vehicle emissions) and industrial emissions
Suburban and rural	Biomass burning (e.g. domestic coal burning), vehicle emissions and industrial emissions
Industrial	Industrial emissions, vehicle emissions and soil dust
Remote coastal areas	Sea salt, vehicle emissions, soil dust, biomass burning

Studies conducted in the Waterberg, Highveld and Vaal Triangle regions of South Africa found that sources such as wood/biomass burning, soil dust, secondary aerosols and domestic combustion were present in all regions, but that industrial, petrochemical and motor vehicle sources were only present in the Highveld and Vaal Triangle regions (Walton, 2021).

The composition of PM_2.5_ may be complex. Carbonaceous aerosol particles form part of PM_2.5_ air pollution and poses harmful effects to public health, environment, agricultural production and visibility (Bisht et al., 2015; Li et al., 2016; Janssen et al., 2011). Carbonaceous aerosols consist of two main types of carbon, organic carbon (OC) and elemental carbon (EC), sometimes referred to as black carbon (BC). EC and BC particulates result from incomplete combustion of substances containing carbon. OC in turn may result from resuspended soil that contains residues from coal-based industrial exhaust, biomass burning, vehicle exhaust, degraded biomass, etc. OC is classified in two groups, namely, secondary organic carbon (SOC) and primary organic carbon (POC). SOC results from heterogeneous chemical reactions during conversion processes (gas to particle) (Wang et al., 2018; Mahilang et al., 2020). BC is distinguished from other carbonaceous aerosols by the fact that it absorbs visible light (Bond et al., 2013). Equivalent black carbon, also known as soot, contains all three carbonaceous aerosol types, namely, EC, BC and OC. Each aerosol type may be compared to soot separately.

Suspended PM_2.5_ travels long distances for hours or days, thus being classified as long-range transport (LRT) pollutants. Backward trajectory modelling is an important tool that is often used to determine the origin of PM_2.5_ sources (Adeyemi et al., 2021; Howlett‑Downing et al., 2022). In the present study, clusters of back trajectories were determined as done in previous studies where four main clusters were identified (Adeyemi et al., 2021; Howlett‑Downing et al., 2022).

Making use of receptor source apportionment models is the most effective approach to source apportionment. These models provide information about source profiles, mass contribution of each profile to PM and number of PM sources. Elements are grouped together by means of correlated concentration trend, with the groups being associated with real-world sources based on source profiles (Mazzei et al. 2008). Anthropogenic sources of PM air pollution in South Africa include motor vehicles, factories, mining, agriculture, coal and biomass burning (wood burning, heating and cooking) (Scorgie et al., 2004). Source apportionment, backward trajectories and trace element composition may all be applied to help locate PM_2.5_ air pollution sources.

The Mangaung municipality (Bloemfontein) in South Africa has no Air Quality Management Plan in place, as required by the national Air Quality Act (South African Air Quality Information System, 2023). This municipality has three air quality stations; however, data are available from only the Pelonomi site (coordinates: −29.139503, 26.241572, 7.6 km from the present sampling site) (South African Air Quality Information System 2023). The aim of the present study was to identify local and distant sources of PM_2.5_ pollution in the Bloemfontein area.

## Materials and methods

Van der Westhuizen et al. (2022) recently reported related sampling, gravimetric and chemical analysis methods. In this study, additional analyses of previously obtained results were done, which include black and organic carbon analyses, determination of long-range transport clusters and pollution sources.

### PM_2.5_ sampling and gravimetric analysis

The climate in Bloemfontein is semi-arid tropical and subtropical steppe (BSk) according to the Köppen climate classification and the seasons experienced are autumn (March to May), winter (June to August), spring (September to November) and summer (December to February).

PM_2.5_ were continually collected over 24-h periods every third day from June 16, 2020 to August 18, 2021, on 37-mm PTFE membrane filters (Zefon International, FL, USA) using GilAir-5 personal air samplers with a flow rate of 4.0 L/min (Sensidyne, Schauenburg Electronic Technologies Group, Mulheim-Ruhr, Germany). The study site was the weather station at the University of the Free State (coordinates −29.1074891, 26.188941; 29°06′27.0″S 26°11′20.2″E) in Bloemfontein, South Africa. As also in previous studies (Adeyemi et al., 2021; Howlett‑Downing et al., 2022), these samplers were again preferred, also being more cost effective than ambient sampling equipment.

The filter samples were weighed at the School of Health Systems and Public Health, University of Pretoria, in batches of 20 before and after sampling. An ultra-micro-balance (Mettler-Toledo XP6) was used under climate-controlled conditions (temperature: 20.1–22.0 °C, relative humidity: 43–54%). Filter samples were stored in a refrigerator at 4 °C.

### Smoke stain reflectometry

Reflectance measurements were performed using an EEL43 reflectometer (Diffusion Systems Ltd. EEL model 43 D) at the School of Health Systems and Public Health, University of Pretoria (Adeyemi et al., 2021; Howlett‑Downing et al., 2022). Light absorption or reflectance of PM_2.5_ collected on filters is a marker for particles produced by incomplete combustion (RUPIOH, 2022). The absorption coefficient (m^−1^ × 10^−5^) for soot was calculated from the reflectance values of each filter sampled.

### Optical transmissiometry

Analyses of BC (measured at 880 nm) and UV-PM (a proxy for organic carbonaceous particulate matter absorbing UV light at 370 nm) (Wichmann et al., 2014) were performed on filter samples using a Model OT21 Optical Transmissometer (Magee Scientific Corp., Berkeley, CA, USA) at the Department of Occupational and Environmental Medicine, Institute of Medicine, Sahlgrenska Academy, University of Gothenburg, Sweden (Adeyemi et al., 2021; Howlett‑Downing et al., 2022). Additional absorption in UV light, due to the presence of organic compounds, indicates the presence of biomass burning (Sandradewi Jet al., 2008; Teich et al. 2017).

### X-ray fluorescence

Energy dispersive X-ray fluorescence (EDXRF) analysis was used to determine the elemental composition of PM_2.5_ (Van der Westhuizen et al., 2022). An XEPOS 5 energy dispersive X-ray fluorescence (EDXRF) spectrometer (Spectro analytical instruments GmbH, Germany) was used at the Atmospheric Science Division, Department of Chemistry and Molecular Biology, University of Gothenburg (Adeyemi et al., 2021; Howlett-Downing et al., 2022). The XRF spectrometer is equipped with palladium and cobalt anodes in four energy ranges: 6–19 keV, >19 keV, 3–6 keV, <3 keV for optimal analytical conditions. The concentrations of 19 elements (Ag, Ba, Br, Ca, Cl, Cr, Cu, Fe, K, Mn, Ni, P, S, Si, Sr, Ti, U, V and Zn) were determined and calculated. The use of a sample changer tray, where each sample is rotated during analysis, overcomes the influence of any uneven distribution of particles on the filter surface. An air filter analysis method was selected in the XRF Analyzer Pro evaluation software and the data were evaluated using a fundamental parameter function. The basic physical principles of XRF are used for calibration and data evaluation.

Calibration was performed monthly by analysing a standard sample supplied by the spectrometer manufacturer. By repeated analysis (*N* = 7) of two randomly selected filters with different mass loadings, the average analytical precision was between 15 and 20% (Howlett-Downing et al., 2022).

### Long-range air mass transport

The geographical origins of air masses travelling through Bloemfontein were used as a proxy for the determination of long-range transport clusters of air pollutants from distant sources (Molnar et al. 2017; Schwarz et al., 2016; Tshehla & Djolov, 2018; Williams et al., 2020). Seventy-two-hour backward trajectories were generated for every day of the 14-month sampling period (June 16 2020 to August 18 2021) using the Hybrid Single Particle Lagrangian Integrated Trajectory (HYSPLIT) program (Tshehla & Djolov, 2018; Williams et al., 2020; Draxler & Rolph, 2003). The Global Centers for Environmental Prediction/National Centers for Atmospheric Research (NCEP/NCAR) web server utilized the global reanalysis meteorological data from the National Oceanic and Atmospheric Administration Air Resource Laboratory (NOAA ARL). Analysis fields (2.5° × 2.5° resolution, 17 vertical levels, 10 000 m AGL) were generated for 6-h intervals (00:00, 06:00, 12:00 and 18:00) and wind fields were linearly interpolated between each interval (Tshehla & Djolov, 2018; Williams et al., 2020; Wichmann et al., 2014). This study used trajectories of three different starting heights (250 m, 500 m and 750 m) and a fixed offset grid factor (250 m). Average 72-h backward daily trajectories were calculated for cluster analysis, as done in previous studies (Adeyemi et al., 2021; Howlett‑Downing et al., 2022). The study period generated a total of 5208 trajectories and was as such used in the clustering analysis which was done for the entire study period. The distance between a trajectory endpoint and the associated cluster mean endpoint was used to drive the clustering algorithm in HYSPLIT (Adeyemi et al., 2021). A total spatial variance (TSV) plot was used to determine the optimum number of clusters (HYSPLIT Tutorial, 2023). Four clusters were established, namely, Mpumalanga (MP), short Indian Ocean (SIO), Northern Cape (NC) and long Atlantic Ocean (LAO).

### Positive matrix factorization (PMF) source apportionment

The source apportionment analysis in this study was performed using the US Environmental Protection Agency (EPA) PMF 5.0 program (PMF 5.^0^, ^2023^), which is widely used to investigate PM_2.5_ source origins (Tshehla & Djolov, 2018; Martins et al., 2016). The multivariate receptor PMF model uses the weighted least squares approach to provide estimated source profiles and contributions (Paatero & Tapper, 1994; Paatero, 1997). The advantage of using PMF analyses is that they can handle incomplete data, such as data with negative values, missing data and data below the limit of detection. However, there were no negative values, missing data or data below the limit of detection in this study (Chueinta et al, 2000, Siregar et al, 2022). Adeyemi et al. (2021) performed similar analyses and found that the best results were obtained using the lowest *Q* value. In addition, they showed that parameters such as concentration and uncertainties are important when performing PMF model analyses. Species were classified according to signal-to-noise ratio (s/n). When the ratio was < 0.1, it was considered ‘poor’; when it was ≥ 2, it was considered ‘strong’, while values between 0.1 and 2 were considered ‘weak’. In the PMF software, PM_2.5_ mass was set as a ‘total variable’ and was therefore ‘weak’ by default (Lee et al., 2011). With this PMF software setting, the software automatically subtracts the mass of all variables from the PM_2.5_ mass and maintains the mass balance. The following settings were used for the base model run: number of base runs (20), selected base run (5), number of seeds (32), number of factors (5) and additional modelling uncertainty (0%). In addition, the *Q*_*true*_ and *Q*_*robust*_ values for base run 5 were 11074.6 and 6410.73, respectively. The bootstrap settings were as follows: number of bootstrap runs (100), number of seeds (32) and *R*^2^ value (0.6). The PMF model was run with five, six and seven factors. Five factors were chosen as this gave the best *Q* value and the factor profiles could be explained by known sources. This method requires interpretation; the source factors are determined using knowledge of the environment and previous studies combined with traffic cluster analyses. The method was validated by estimating uncertainty and stability using bootstrap and error of displacement calculations. Of course, due to changing weather patterns and the specific source allocations made, potential biases in this method cannot be excluded.

### Descriptive statistics

Statistical analyses were performed with STATA, version 15 (Adeyemi et al., 2022; STATA, 2023). As reported by Van der Westhuizen et al. (2022), according to the Shapiro–Wilk’s test, PM_2.5_ levels and its chemical component concentrations did not show normal Gaussian distributions; hence, non-parametric tests were applied. Kruskal–Wallis tests were conducted to investigate whether PM_2.5_ concentrations and the identified PMF sources differed significantly across seasons and between the four transport clusters: MP, SIO, NC and LAO representing different geographical air mass origins.

## Results

### Mass and composition of PM_2.5_

**Fig. 1:**
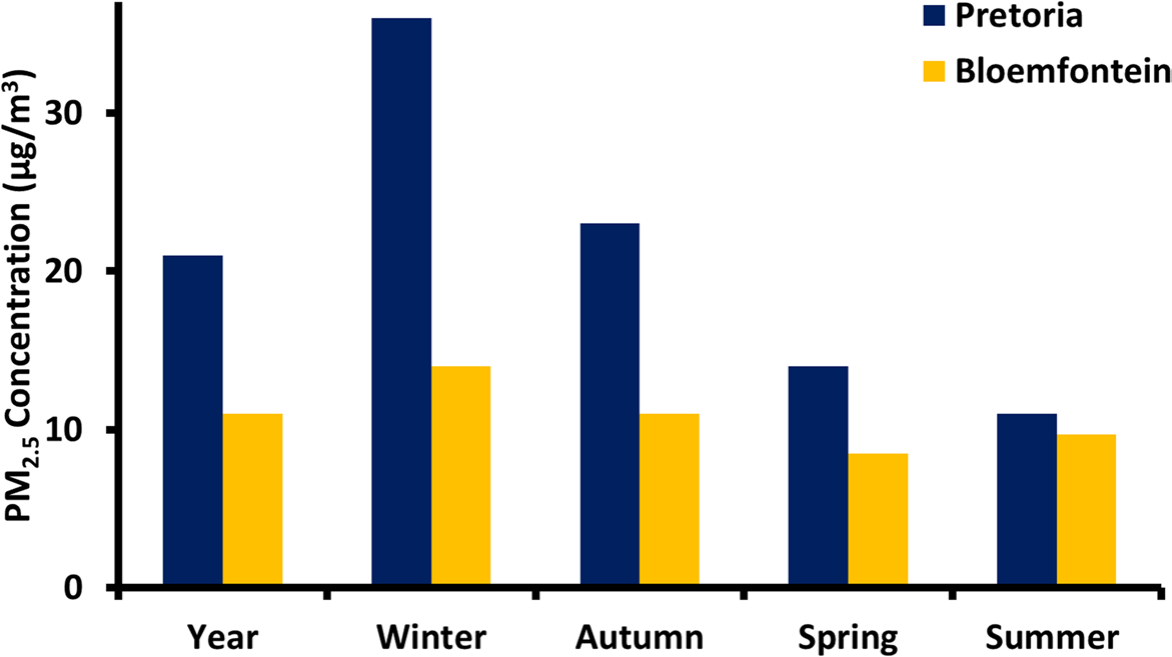
Comparison between the PM_2.5_ concentrations of Bloemfontein and Pretoria

**Fig. 2:**
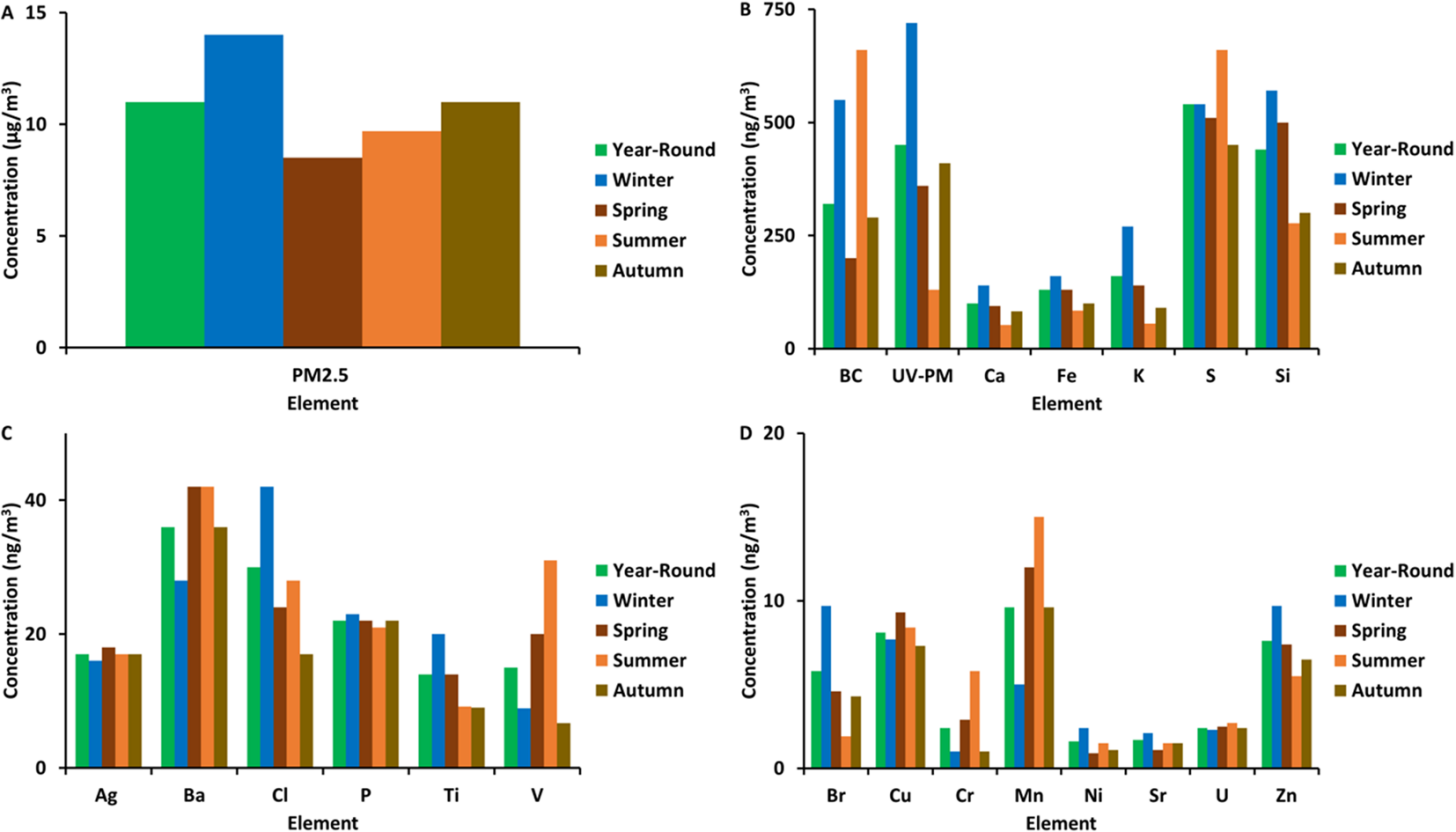
Seasonal mean concentration variations of elements, as listed in Table 2

The mean PM_2.5_ concentration during the study period was 11 µg/m^3^ (ranging from 0.52 to 51 µg/m^3^) (Table 1), which exceeded the annual World Health Organization (WHO) guideline (5 µg/m^3^), but not the annual South African National Ambient Air Quality Standard (20 µg/m^3^) (WHO, 2021; Department of Environment Affairs, 2012). Similar studies were done elsewhere in South Africa; two studies were performed in Pretoria (Adeyemi et al., 2021; Howlett‑Downing et al., 2022), reporting average PM_2.5_ concentrations of 21 and 24 µg/m^3^, respectively. Studies in Cape Town, Limpopo and Thohoyandou reported related values of 13, 12 and 11 µg/m^3^ (Tshehla & Djolov, 2018; Williams et al., 2020; Novela et al., 2020). The PM_2.5_ concentration of the present study is half of what was measured in Pretoria, but the seasonal behaviour is the same in the two studies (Adeyemi et al., 2021). A comparison between the PM_2.5_ concentration level of Bloemfontein and Pretoria is illustrated in Fig. 1. The daily PM_2.5_ concentrations never exceeded the daily South African National Ambient Air Quality Standard (40 µg/m^3^) but did exceed the daily WHO guideline (15 µg/m^3^) on 28 of the 145 sampling days (19%). The average soot concentration and absorption coefficient was 1.2 µg/m^3^ (ranging from 0.86 to 2.3 µg/m^3^) and 2.2 × 10^−5^ m^−1^ (ranging from 1.5 × 10^−5^ to 4.1 × 10^−5^ m^−1^) (Table 2). The average BC concentration was 0.32 µg/m^3^ during the study period (ranging from 0.0006 to 2.3 µg/m^3^), while the UV-PM (organic carbon) concentration was 0.45 µg/m^3^ (ranging from 0.0022 to 2.3 µg/m^3^). The trace elements, black and organic carbon concentrations used in the PMF analyses are reported in Table 2 and seasonal mean concentration variations of each element are graphically represented in Fig. 2. The reported data for the soot analysis were excluded, since it is a combination of BC, UV-PM and other carbonaceous aerosols. The most abundant trace elements were S, Si, K, Fe, Ca, Ba and Cl, as seen in Fig. 2B and C.

**Table 2:** Descriptive statistics of PM_2.5_, soot, BC, UV-PM and trace elemental concentrations in Bloemfontein, South Africa, during June 16, 2020 to August 18, 2021 (145 sampling days, 180 samples, 35 duplicates). PM_2.5_, BC, UV-PM and soot concentrations are reported in µg/m^3^ and trace element concentrations are in ng/m^3^

Species	Full study (*n* = 180)		Winter (JJA) (*n* = 66)		Spring (SON) (*n* = 37)		Summer (DJF) (*n* = 38)		Autumn (MAM) (*n* = 39)	
	Mean	Range	Mean	Range	Mean	Range	Mean	Range	Mean	Range
PM_2.5_	11	0.5–51	14	1.8–33	8.5	0.52–25	9.7	1.7–51	11	1.4–33
BC	0.32	0.0006–2.3	0.55	0.0006–2.3	0.2	0.0034–1.1	0.66	0.0014–0.23	0.29	0.001–1.9
UV-PM	0.45	0.0022–2.3	0.72	0.02–2.3	0.36	0.038–1.2	0.13	0.0022–0.36	0.41	0.063–1.6
Soot	1.2	0.81–2.4	1.4	1.3–2.4	1.1	1.1–1.6	1.0	1.1–1.2	1.2	1.1–1.7
Ag	17	11–37	16	11–37	18	11–36	17	11–33	17	11–37
Ba	36	0.3–67	28	0.3–67	42	12–63	42	18–63	36	2.3–64
Br	5.8	0.4–47	9.7	0.4–47	4.6	0.4–14	1.9	0.4–4.4	4.3	0.4–20
Ca	100	0.4–390	140	27–390	94	15–240	52	0.4–130	82	15–230
Cu	8.1	2.9–17	7.7	2.9–16	9.3	4.2–17	8.4	2.9–15	7.3	2.9–15
Cl	30	0.8–260	42	0.8–260	24	4.9–120	28	1.0–220	17	2.8–34
Cr	2.4	0.5–46	1.0	0.5–24	2.9	0.5–35	5.8	0.5–46	1.0	0.5–3.7
Fe	130	7.3–610	160	7.3–380	130	23–340	84	16–610	100	30–240
K	160	6.6–830	270	23–830	140	6.6–520	55	8.2–180	90	22–550
Mn	9.6	0.2–40	5.0	0.2–23	12	0.7–40	15	0.7–37	9.6	0.5–24
Ni	1.6	0.2–25	2.4	0.2–16	0.9	0.8–0.9	1.5	0.8–25	1.1	0.8–5.8
P	22	0.3–51	23	1.1–51	22	0.3–36	21	13–30	22	4.0–44
S	540	5.0–3400	540	21–3400	510	9.6–2800	660	5.0–1900	450	30–1700
Si	440	15–1900	570	24–1700	500	46–1900	277	15–1700	300	92–790
Sr	1.7	0.0–7.2	2.1	0.1–5.6	1.1	0.0–3.2	1.5	0.0–3.9	1.5	0.0–7.2
Ti	14	0.0–76	20	2.3–58	14	2.0–76	9.2	0.0–64	9.0	0.0–34
U	2.4	0.0–7.3	2.3	0.2–7.3	2.5	0.0–4.3	2.7	0.1–4.3	2.4	0.2–6.9
V	15	0.6–160	8.9	0.6–110	20	0.6–160	31	0.6–160	6.7	0.6–81
Zn	7.6	2.0–33	9.7	2.0–30	7.4	2.8–33	5.5	2.8–16	6.5	2.0–16

### Transport clusters and potential source areas

**Fig. 3:**
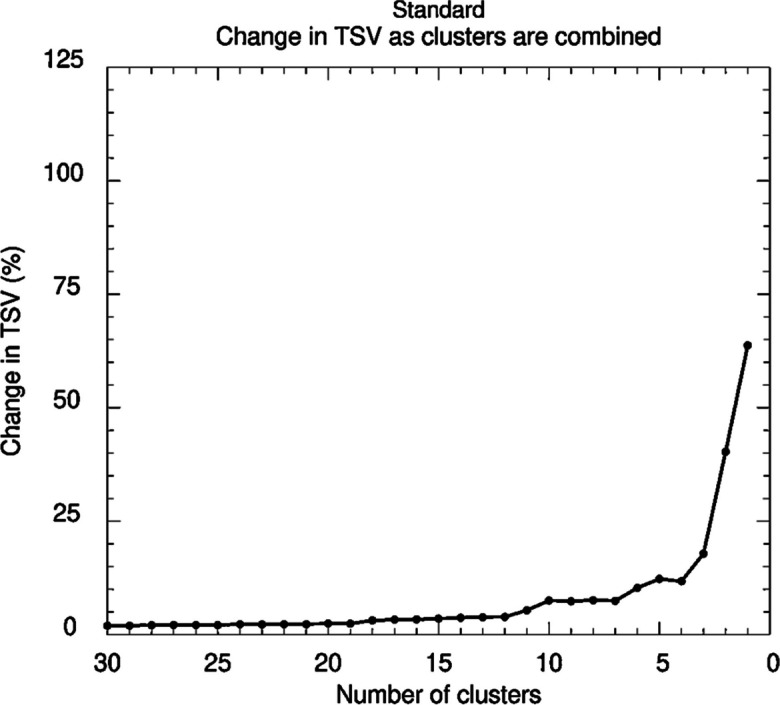
TSV plot for estimating the optimum number of clusters

The TSV plot in Fig. 3 suggests four, five or six clusters, where five and six resulted in overlapping (see Figure S1), thus 4 clusters were chosen as the optimum number of clusters.

**Fig. 4:**
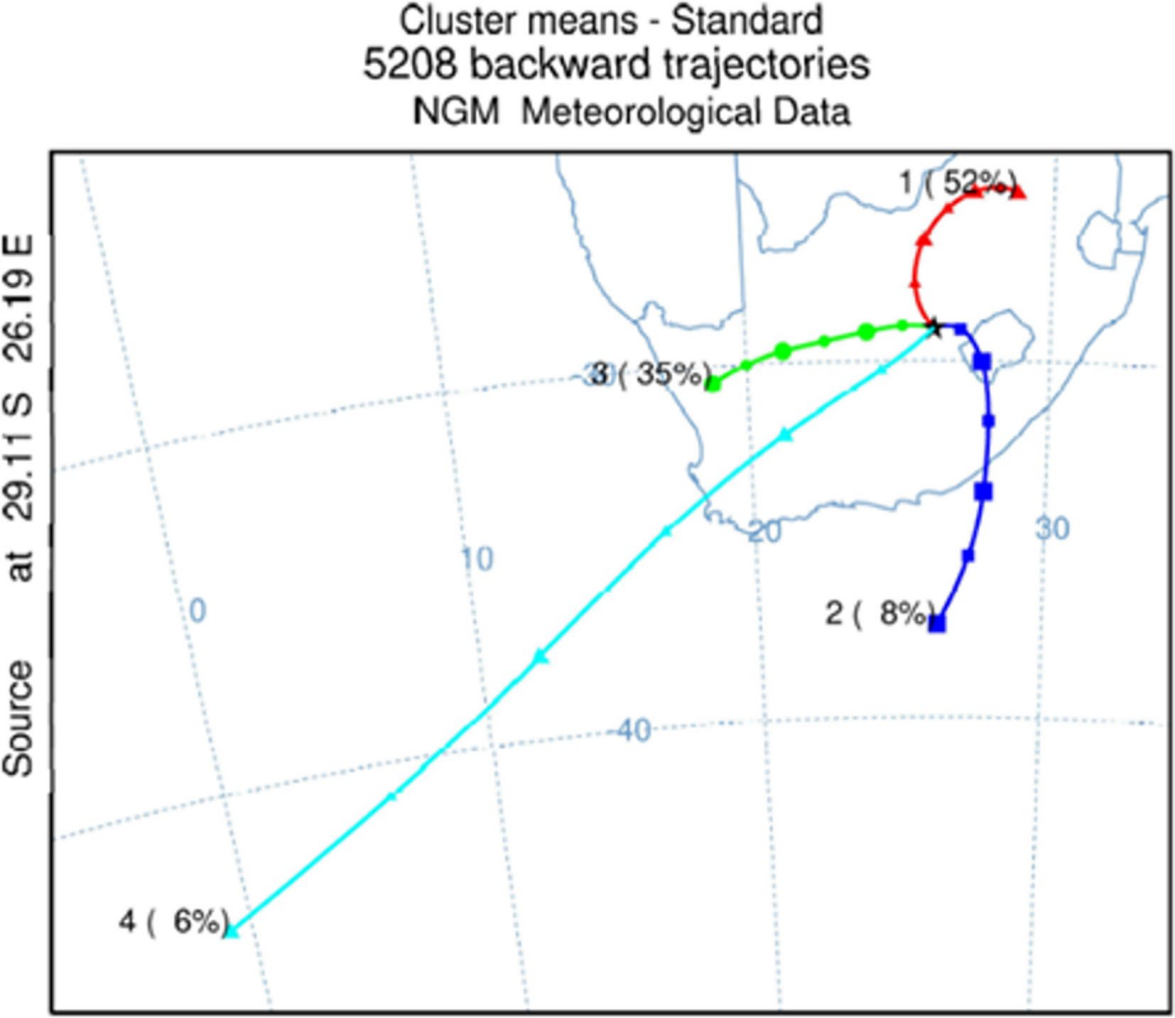
Geographical origin of air masses that passed through Bloemfontein from June 16, 2020 to August 18, 2021, presented as backward trajectory cluster means, calculated for the starting heights 250 m, 500 m and 750 m

Four back trajectory cluster pathways were identified from the 5208 individual backward trajectories, see Fig. 4. Cluster 1 is the Mpumalanga province (MP) cluster (52% of the mass concentration), cluster 2 is the short Indian Ocean (SIO) cluster (8%), cluster 3 is the Northern Cape province (NC) cluster (35%) and cluster 4 is the long Atlantic Ocean (LAO) cluster (6%) travelling over the Western Cape province (WC).

The MP cluster travels over the Gauteng (GP), North West (NW) and Free State provinces (FS). GP is known for industrial activities and it also has the highest population in South Africa (SA). NW, FS and MP are known for its mining and agriculture. The SIO cluster travels over the Eastern Cape province (EC) and Lesotho (L). The EC is known for agricultural and industrial activities, while Lesotho is known for manufacturing, mining and agricultural activities. The NC clusters travel over the NC and the FS, which are provinces known for its mining and agriculture. The LAO cluster travels over the Western Cape province (WC), the province that is known for its industries and agriculture. The SIO cluster contribution reflects an oceanic contribution.

### Positive matrix factorization source apportionment

**Fig. 5:**
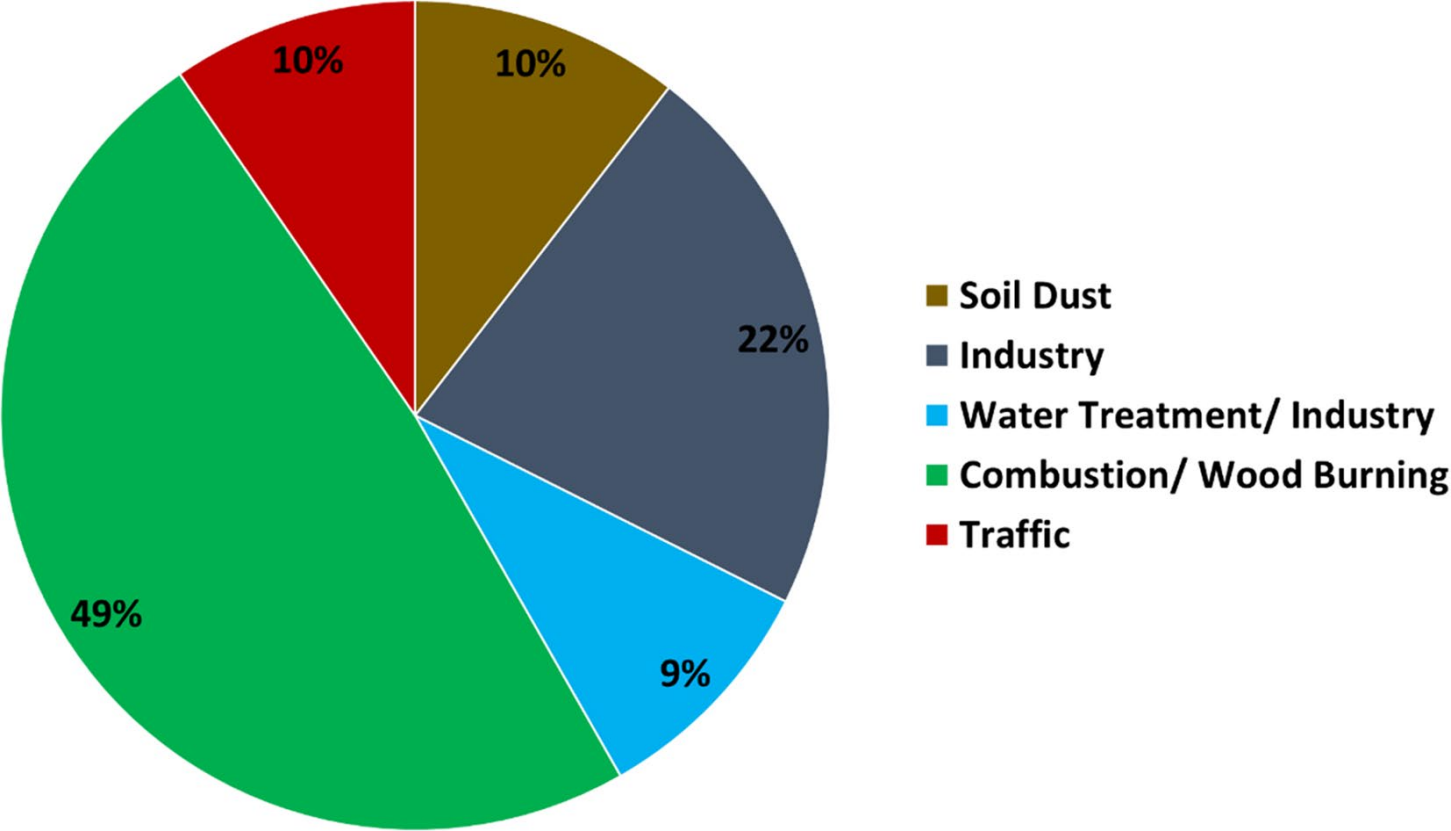
Percentage factor contribution of each PMF factor

Elemental contributions to the source profiles were determined by the s/n ratios of the elements. Eighteen elements (Ag, Ba, Br, Ca, Cl, Cr, Cu, Fe, K, Mn, Ni, S, Si, Sr, Ti, U, V and Zn) had strong or weak ratios and were included in the profiling together with PM_2.5_, BC and UV-PM, while the remaining one element with bad s/n ratio (P) was excluded from the analyses. Five factors and their percentage contributions were identified, namely, combustion/wood burning (49%), industry (22%), soil dust (10%), traffic (9.6%) and water/water treatment/industry (9.4%), see Fig. 5. Reported mass percentage contributions of each specie/element to each factor are shown in Table 3 and Figure S2. The concentrations during the study period (145 days) and the seasonality of each factor are reported in Table 4 and Figure S3. A Kruskal–Wallis test confirmed that all values were different during seasons and statistically significant (*p* < 0.05), except the water treatment/industry factor (*p* > 0.05). The latter indicates that combustion/wood burning, water treatment/industry, soil dust and traffic factors are seasonal and the industry factor is ubiquitous. Winter and spring seasons have higher concentrations in each factor, as seen in Figure S3. The only factor that has similar concentrations year-round is the industry factor. Autumn has the highest industry factory contribution and the lowest contribution in all the other factors.

**Table 3:** Mass percentage contribution of each specie/element to each PMF factor

Specie	Soil dust	Industry	Water treatment/industry	Combustion/wood burning	Traffic
PM_2.5_	7.3	20	11	54	7.5
BC	0.0	0.0	0.0	70	30
UV-PM	0.8	4.3	0.0	55	40
Si	63	9.1	7.6	18	1.6
S	0.8	84	0.0	15	0.5
Cl	3.0	0.0	86	11	0.0
K	16	2.8	5.4	65	9.9
Ca	17	0.0	2.6	26	54
Ti	60	13	6.9	19	0.8
V	8.3	28	23	3.0	38
Cr	6.8	28	30	0.0	35
Mn	7.0	58	0.0	0.0	35
Fe	38	6.4	4.8	20	30
Ni	7.5	16	42	11	23
Cu	22	16	10	9.7	42
Zn	0.0	16	5.6	31	47
Br	2.2	0.0	4.5	61	32
Sr	15	10	6.4	21	47
Ag	16	20	11	7.7	45
Ba	23	31	11	0.0	35
U	0.0	5.5	53	11	31

**Table 4:** The factor concentrations over the study period and the seasonality of each factor. Kruskal–Wallis tests confirmed that all the values are different during seasons and statically significant (*p* < 0.05), except the industry factor (*p* > 0.05). Concentrations are reported in µg/m^3^ over 145 sampling days, 180 samples and 35 duplicates

	Full study		Autumn (MAM)		Winter (JJA)		Spring (SON)		Summer (DJF)	
Days observed	145		31		54		30		30	
	Mean	Range	Mean	Range	Mean	Range	Mean	Range	Mean	Range
Combustion/wood burning	6.5	0–55	0.5	0–6.0	12	0–55	8.4	0–31	1.6	0–18
Industry	3.9	0–22	4.3	0–13	3.9	0–22	3.2	0–18	4.0	0–21
Soil dust	1.7	0–12	0.64	0–1.3	1.8	0–7.7	2.3	0–7.1	1.9	0–12
Traffic	1.5	0–6.8	1.1	0–1.9	1.9	0–6.8	1.8	0–4.5	1.1	0–2.3
Water treatment/industry	1.6	0–13	1.2	0–12	1.9	0–13	1.6	0–6.7	1.4	0–12

Elemental contribution to each factor can be sorted into three groups, namely, main elemental contribution (>50%), moderate elemental contribution (20–50%) and low elemental contribution (5–20%). Table 5 reports the elemental contribution to each factor.

**Table 5:** Contribution of elements to each factor sorted into main, moderate and low contribution groups

Factor	Main elemental contribution (>50%)	Moderate elemental contribution (20–50%)	Low elemental contribution (5–20%)
Combustion/wood burning	PM_2.5_, BC, UV-PM, K, Br	Ca, Fe, Zn, Sr	Si, S, Cl, Ti, Ni, Cu, Ag, U
Industry	S, Mn	PM_2.5_, V, Cr, Ag, Ba	Si, Ti, Fe, Ni, Cu, Zn, Sr, U
Soil dust	Si, Ti	Fe, Cu, Ba	PM_2.5_, K, Ca, V, Cr, Mn, Ni, Sr, Ag
Traffic	Ca	BC, UV-PM, V, Cr, Mn, Fe, Ni, Cu, Zn, Br, Sr, Ag, Ba, U	PM_2.5_
Water treatment/industry	Cl, U	V, Cr, Ni	PM_2.5_, Si, K, Ti, Zn, Sr, Ag, Ba

## Discussion

From Table 2, it may be seen that soot and UV-PM show the highest concentrations in winter and the lowest concentrations in summer. The same was observed with the soot analyses done in a previous study (Van der Westhuizen et al., 2022). The BC concentration on the other hand was high in winter, but the summer concentration was high as well. This indicates a possible difference in source contribution concentrations of inorganic carbonaceous species in Bloemfontein. On the other hand, a good linear correlation exists between soot, BC and UV-PM (Figures S4 – S6), illustrating that BC and UV-PM make up a considerable soot contribution, which indicates possible similarity in sources. Correlations of soot vs BC, soot vs UV-PM and BC vs UV-PM were 0.83, 0.88 and 0.93, respectively. BC and UV-PM are emitted from combustion sources like biomass and coal burning, which are big local contributors of air pollution in Bloemfontein.

This is confirmed by the high contribution of BC and UV-PM to the combustion factor in the PMF analysis. More than 50% of the BC and UV-PM concentration can be found in the combustion factor, see Table 5. According to the Spearman correlation analysis (Table S1), BC has statistically significant correlations with many of the reported elements/species in the PM_2.5_ content analyses. The Spearman correlation for UV-PM also shows statistically significant correlations (Table S1) with many elements, but not with all the elements correlated with BC. This shows a possible difference in sources of the two carbon species. A study in Pretoria (Adeyemi et al., 2021) indicated similar results for BC and UV-PM; the main difference was the correlations between BC and PM_2.5_ being strong in the current study. The difference is attributed to differences in local and regional sources between the two sites, see Table 6.

**Table 6:** Spearman correlations between BC, UV-PM and PM_2.5_ for the Bloemfontein (BFN) and Pretoria (PTA) (Adeyemi et al., 2021) sites. All the correlations have positive significant correlations (*p* < 0.5)

	BFN	PTA
PM_2.5_ with BC	0.43	0.76
PM_2.5_ with UV-PM	0.51	0.76
BC with UV-PM	0.95	0.98

Of the four back trajectory clusters determined in the HYSPLIT analysis, the MP cluster (Fig. 4), which travels over GP, NW, FS and MP, was the most prominent cluster. The clusters can be grouped into two main classes. The trajectories of clusters MP and NC show air masses moving over land, while the SIO and LAO clusters partly travel over oceanic areas, which may influence the source profiles. The areas over which the air masses move have more or less different characteristics, the influence of which can be seen in deeper analysis of the trajectories and filter samples collected at different times.

Combination of the back trajectory clusters and PMF source profiles presents a better understanding of sources contributing to air pollution. The factors contributing to PM_2.5_ in Bloemfontein are combustion/wood burning, industry, soil dust, traffic and water treatment/industry. A study in Pretoria identified contributing factors such as fossil fuel combustion, soil dust, secondary sulphur, vehicle exhaust, road traffic, base metal/pyrometallurgical and coal combustion (Adeyemi et al. 2021). As mentioned above, studies conducted in the Waterberg, Vaal Triangle and Highveld regions of South Africa found soil dust, wood/biomass burning, secondary aerosols and domestic combustion sources to be ubiquitous. Petrochemical, industrial and motor vehicle sources were present only in the Highveld and Vaal Triangle regions (Walton, 2021).

The summary for the whole study period with the factor contribution for each cluster is described in Table 7, illustrating the contribution to total PM_2.5_ from all sectors to be similar. Differences can be seen in the contribution to the five source factors. Air masses in the MP sector contribute mainly to the combustion/wood burning and industrial factors. The industrial factors are mainly influenced by the energy and mining industries. These air masses pass over parts of South Africa where mining activity is high, which is reflected in the factor contribution. The contribution of the SIO sector to the mining/industry factor is expected as the air masses pass over regions with intense industrial and mining activity. The more even contribution of the NC sector to all factors indicates a greater diversity of activities in this sector. Wind from the LAO sector is the main contributor to the water/wastewater/industry and combustion factors. The air masses pass over large areas of land, contributing to the combustion factor. As the trajectory for this sector is long, it has higher wind speeds than the other sectors and may therefore contribute some oceanic influence to the water/water treatment/industry factor.

**Table 7:** Summary of the back trajectory clusters of the whole study period, the factor contribution of each factor and PM_2.5_ concentration contribution to each cluster. All concentrations are in µg/m^3^

	Cluster (days)	Total PM_2.5_	Combustion/wood burning	Industry	Soil dust	Traffic	Water treatment/industry
1	MP (79)	12	8.5	3.9	1.7	1.6	1.7
2	SIO (9)	7.8	2.1	4.5	0.98	1.1	0.87
3	NC (49)	11	4.6	4.0	1.8	1.5	1.5
4	LAO (8)	12	2.7	1.9	1.3	1.5	1.1

The seasonality of each transport cluster and factor contributors are illustrated in Figure S7. In the winter season, a high contribution to PM_2.5_ can be observed in most clusters. The winter season is characterized by dry and cold air with mean temperatures ranging between −2 and 15 °C. The dominating wind direction is north, and we thus expect influence from the industrial activities in GP as well as mining and agriculture in the NW and FS provinces. In this wind sector, the MP cluster is dominating, but we see influence of the dry seasonal conditions in the other clusters as well. For the other land-based cluster (NC), the air masses pass over the NC and FS provinces and should have an influence from agriculture and mining activities. The northerly wind is dominating year-round, but in the autumn season, Bloemfontein experiences winds from the east and south as well, which should introduce a difference between the MP and NC clusters, see Figure S7. In Figure S7 and Table 8, it is seen that the influence of industry activities is higher in the summer when the influence is associated with the MP cluster. The SIO cluster adds influence not only from the Indian Ocean, but also from human activities in the EC and Lesotho. The winds should come from east and south, like the cluster shows, during spring and summer seasons. The LAO cluster had the lowest number of contributing days (8) and it was the only cluster that reported no observations in summer and spring seasons.

**Table 8:** Seasonality of the MP, SIO, NC and LAO clusters, and factorial contribution to the cluster

	Cluster (Obs)	Seasons (Obs)	Combustion/wood burning	Industry	Soil dust	Traffic	Water treatment/industry
1	MP (79)	Autumn (13)	0.90	3.9	0.68	1.2	1.7
		Winter (30)	16	4.0	1.8	1.9	1.9
		Spring (16)	11	3.2	2.6	1.8	1.4
		Summer (20)	0.69	4.2	1.7	1.3	1.8
2	SIO (9)	Autumn (3)	0.02	4.7	0.90	1.1	0.86
		Winter (4)	3.9	5.8	1.0	1.1	0.73
		Spring (1)	2.9	0.56	1.2	0.85	0.65
		Summer (1)	0.74	2.3	1.1	1.4	1.7
3	NC (49)	Autumn (13)	0.21	4.9	0.48	1.0	0.74
		Winter (14)	8.0	4.0	2.2	2.3	2.4
		Spring (13)	5.9	3.5	2.1	1.7	1.9
		Summer (9)	3.6	3.6	2.4	0.72	0.63
4	LAO (8)	Autumn (2)	0.82	3.2	1.1	1.2	0.79
		Winter (6)	3.4	1.4	1.4	1.5	1.2
		Spring (0)					
		Summer (0)					

The major PMF factor representing combustion/wood burning processes accounts for 49% of the analysed mass and contains elements from different combustion sources. This factor is mainly influenced by biomass combustion. Biomass sources include open fires, wood burning, with fossil fuel combustion and vehicle emission sources also included in this factor. BC, UV-PM, Br and K are typical elemental tracers for biomass combustion. Zn and smaller amounts of BC are elemental components in exhaust and in non-exhaust vehicle emissions. Zn originates from additives in oil lubricants and is also a component in tires. Cu and smaller amounts of U are elemental components of fossil fuel burning. The high contribution of PM_2.5_, BC and UV-PM to the combustion factor indicates a certain influence from non-complete combustion. That is most likely from biomass burning, but can also originate from coal burning sources in South Africa, including power stations, of which 80% are located in MP and the FS regions. This factor increases during the winter season due to domestic heating. The seasonality of this factor is seen in Table 4 (Figure S3) and the seasonality per cluster is illustrated in Figure S7. In the study in Pretoria by Adeyemi et al. (2021), a combustion factor was found to be the dominating PMF factor, but in the present study the combustion factor is mixed compared to three factors, including combustion as indicated in studies by Adeyemi et al. (2021) and Walton (2021).

The industry contribution (22%) falls under the top 4 factors that are causing air pollution in Bloemfontein. The two main elements contributing to this factor was S (84%) and Mn (58%). Secondary S aerosols are formed by gaseous sulphur compounds caused by industries such as the pulp and paper industry, power plants, phosphate industry and metal smelting production. Power generation in South Africa mainly consists of coal and fossil fuel burning. Mn in South Africa represents ca 75% of global resources. Mn also plays an important role in the steel production processes, which are also huge in South Africa. The mining industry influences this factor substantially, for which the NC and MP provinces are known. The seasonality of this factor is reported in Table 4 (Figure S3) and the seasonality per cluster is reported in Figure S7 (Adeyemi et al. 2021 and Walton, 2021).

Soil dust (10%) is wind-blown dust and is linked to mineral dust (Tshehla & Djolov, 2018). Soil dust is marked by Si, Ti and Fe and the main tracers for mineral dust are Si and Ti (Adeyemi et al.^2021^; Almeida et al., 2020). The lack of S and Zn is the distinguishing factor between mineral dust and resuspended dust (Adeyemi et al., 2021). Higher concentrations of this factor is found in the winter and spring seasons, where the FS is known for its wind storms in the months of August and September, which explains the higher concentrations in the winter and spring seasons. The seasonality of this factor is reported in Table 4 (Figure S3) and the seasonality per cluster is reported in Figure S7.

As for traffic (9.6%), this factor consists of a combination of vehicle exhaust and road traffic, where some elements could be linked to base metal/pyrometallurgical tracers such as V (38%), Cr (35%), U (31%), Fe (30%) and Ni (23%). Ferrochromium smelters are linked to Fe and Cr (Adeyemi et al. 2021; Venter et al., 2017), while Ni and V are linked to oil combustion makers and base metal smelter refining processes (Lee et al., 2011; Moreno et al., 2010; Wang et al., 2009). One of the main influencing components of this factor is vehicle exhaust, where tracers include Zn (47%), Cu (42%) and smaller amounts of BC (30%) and UV-PM (40%). Zn and Cu are linked to lubricating oil, while the presence of carbonaceous species is linked to tail pipe emissions (Adeyemi et al. 2021). Road traffic is characterized by a wide variety of elements; some traces include Ca (54%) and Ba (35%). The high Ca concentration may be due to construction, which is high in Ca-dust. The seasonality of this factor is reported in Table 4 (Figure S3) and the seasonality per cluster is reported in Figure S7.

**Fig. 6:**
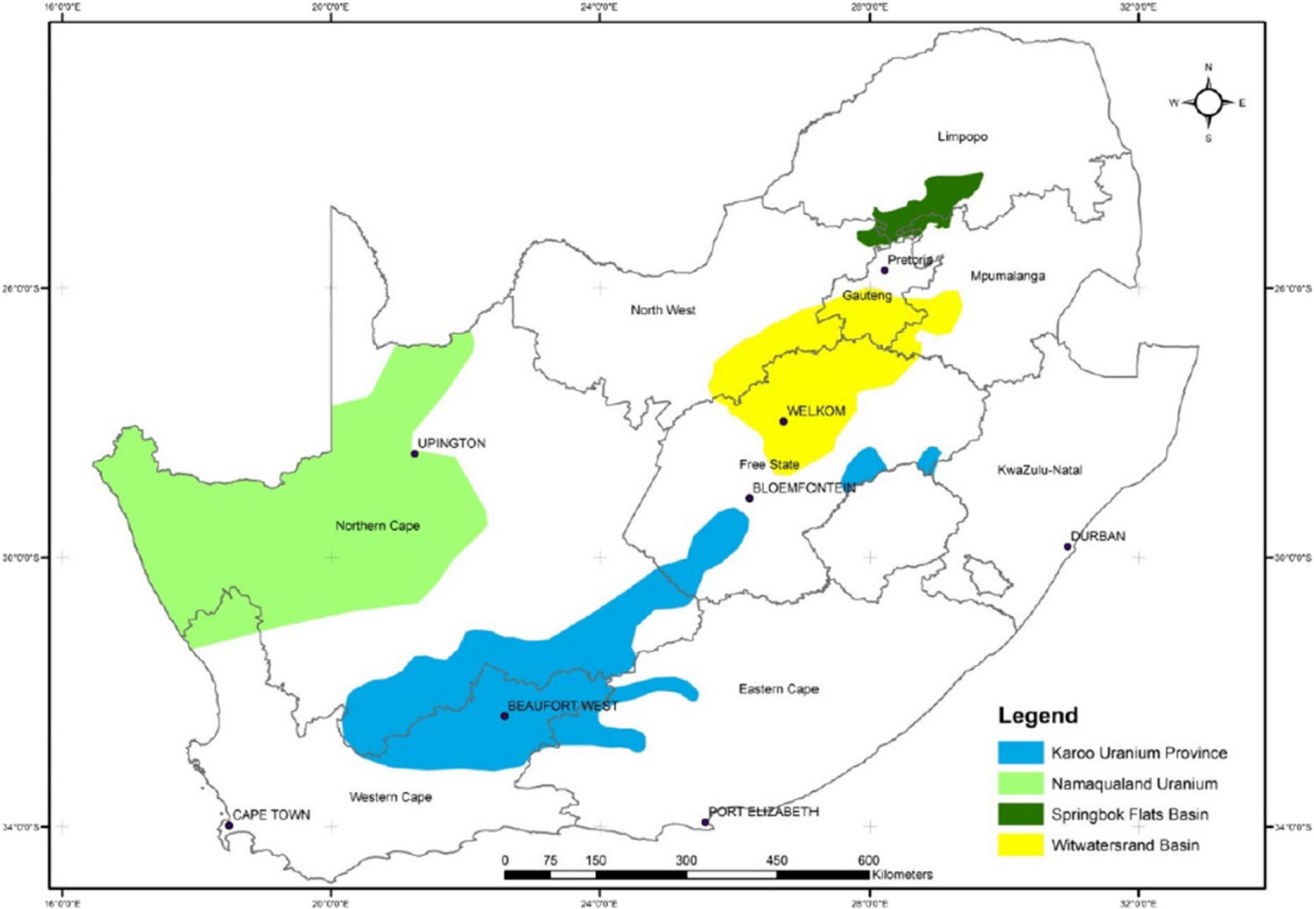
Uranium deposits across South Africa (Kenan & Chirenje, 2016)

The main tracers in the water treatment/industry (9.4%) factor are Cl (86%) and U (53%). South Africa is known for its many residential and sport swimming pools, where Ca(OCl)_2_ is extensively used as a source of Cl_2_ for water clearing purposes. In large water works directly outside towns and cities, the same chemical is also used for municipal water purification. Cl_2_ in combination with Ni, V, Cr and U are also linked to industrial sources. In samples associated with the LAO wind sector, some minor contribution of Cl in sea salt can be expected (Walton, 2021). The NC, WC, MP, GP and FS are known for U mining (U deposits, see Fig. 6) (Kenan & Chirenje, 2016). Confirmation of the U industry contribution can be seen in the comparison of the location of the U sites to the backward trajectories. The seasonality of this factor is reported in Table 4 (Figure S3) and the seasonality per cluster is reported in Figure S7.

## Conclusion

Based on the content analysis of PM_2.5_ and positive matrix factorisation, it can be concluded that the main contribution in Bloemfontein is from biomass and coal combustion. Long range back trajectories were also used in this study to assist in source identification. From the evaluation of the trajectories, it is concluded that due to the similarity of human activities in nearby regions of South Africa, it is difficult to distinguish between local and distant sources. The sources identified in this study have a more regional profile, suggesting the need for further investigation. A comparison of PM_2.5_ concentrations in Bloemfontein and Pretoria shows that PM_2.5_﻿ pollution is worse in Pretoria, where there is certainly room for improvement in air quality. One way to achieve this could be through the use of green energy such as solar and wind power. The sun is a virtually limitless natural resource. By harnessing it efficiently, the impact of thermal power plants and their pollutants on air quality can be dramatically reduced. In addition, the use of open fires should be limited, further reducing the impact of combustion on air quality. This study focused on the sources of PM_2.5_ in Bloemfontein. With the knowledge gained from this study, future studies can be undertaken that focus on the health impacts of each source on human life. This will help to determine the impact of specific industries on air quality and human well-being.

### Supplementary Information

Below is the link to the electronic supplementary material.

Supplementary file1 (DOCX 1129 KB)

## Data Availability

Data generated or analysed during this study are included in this published article and its supplementary information file. Additional data are available from the corresponding author on reasonable request.
