# Supplementary material for: Source apportionment of fine atmospheric particles in Bloemfontein, South Africa, using positive matrix factorization
Source: Environ Monit Assess. 2024 Jan 23;196(2):188. doi: 10.1007/s10661-023-12293-4 (PMC10806018; doi:10.1007/s10661-023-12293-4)
Supplement: Supplementary file 1 — Supplementary file1 (DOCX 1129 KB) [file 10661_2023_12293_MOESM1_ESM.docx]

Source apportionment of fine atmospheric particles in Bloemfontein, South Africa, using positive matrix factorization

Deidré van der Westhuizen^a^, Chantelle Howlett-Downing^b^, Peter Molnár^c^, Johan Boman^d*^, Janine Wichmann^b^* and Karel G. von Eschwege ^a^*

^a. Department of Chemistry, PO Box 339, University of the Free State, Bloemfontein, 9300, South Africa;^ [^didivdwesthuizen@gmail.com^](mailto:didivdwesthuizen@gmail.com) ^, veschwkg@ufs.ac.za^

^b. School of Health Systems and Public Health, Faculty of Health Sciences, University of Pretoria, Pretoria, South Africa;^ [^howlett@gmail.com^](mailto:howlett@gmail.com) ^,^ [^janine.wichmann@up.ac.za^](mailto:janine.wichmann@up.ac.za)^.^

^c. Department of Occupational and Environmental Medicine, Institute of Medicine, Sahlgrenska Academy, University of Gothenburg, Gothenburg, Sweden;^ [^peter.molnar@amm.gu.se^](mailto:peter.molnar@amm.gu.se)^.^

^d. Department of Chemistry and Molecular Biology, Atmospheric Science Division, University of Gothenburg, Gothenburg, Sweden;^ [^johan.boman@gu.se^](mailto:johan.boman@gu.se)^.^

^* Correspondence: JB: johan.boman@gu.se; Tel.:^ [^+46-31-786 00 00^](mailto:+46-31-786%2000%2000)^, KGvE: vEschwKG@ufs.ac.za; Tel.: +27-51-4012923/2194, JW: janine.wichmann@up.ac.za, Tel.: +27-12-356-3259.^


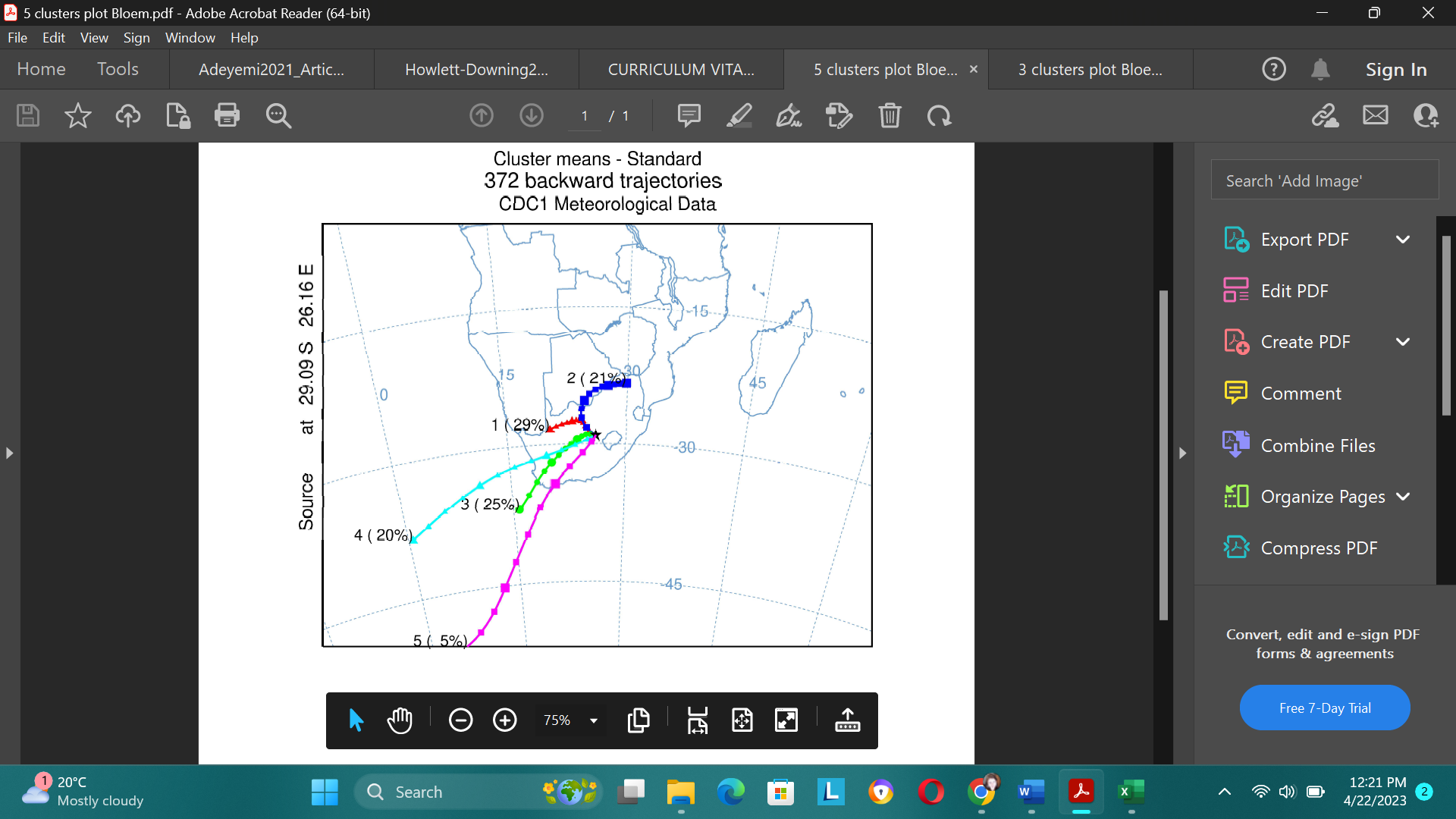

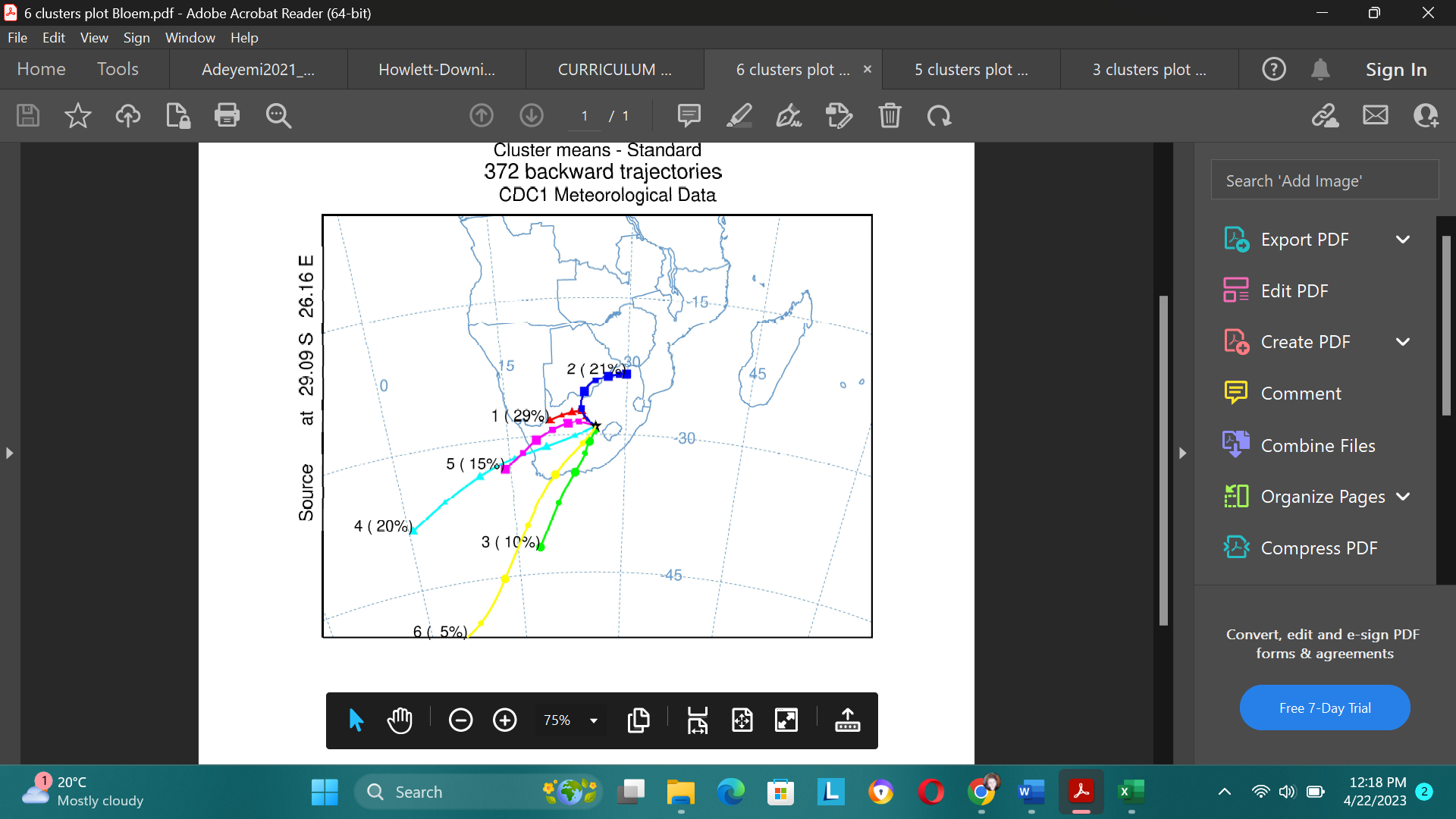


Figure S1 Geographical origin of air masses that pass-through Bloemfontein, presented as backward trajectory cluster means, showing the five (*left*) and six cluster pathways (*right*).


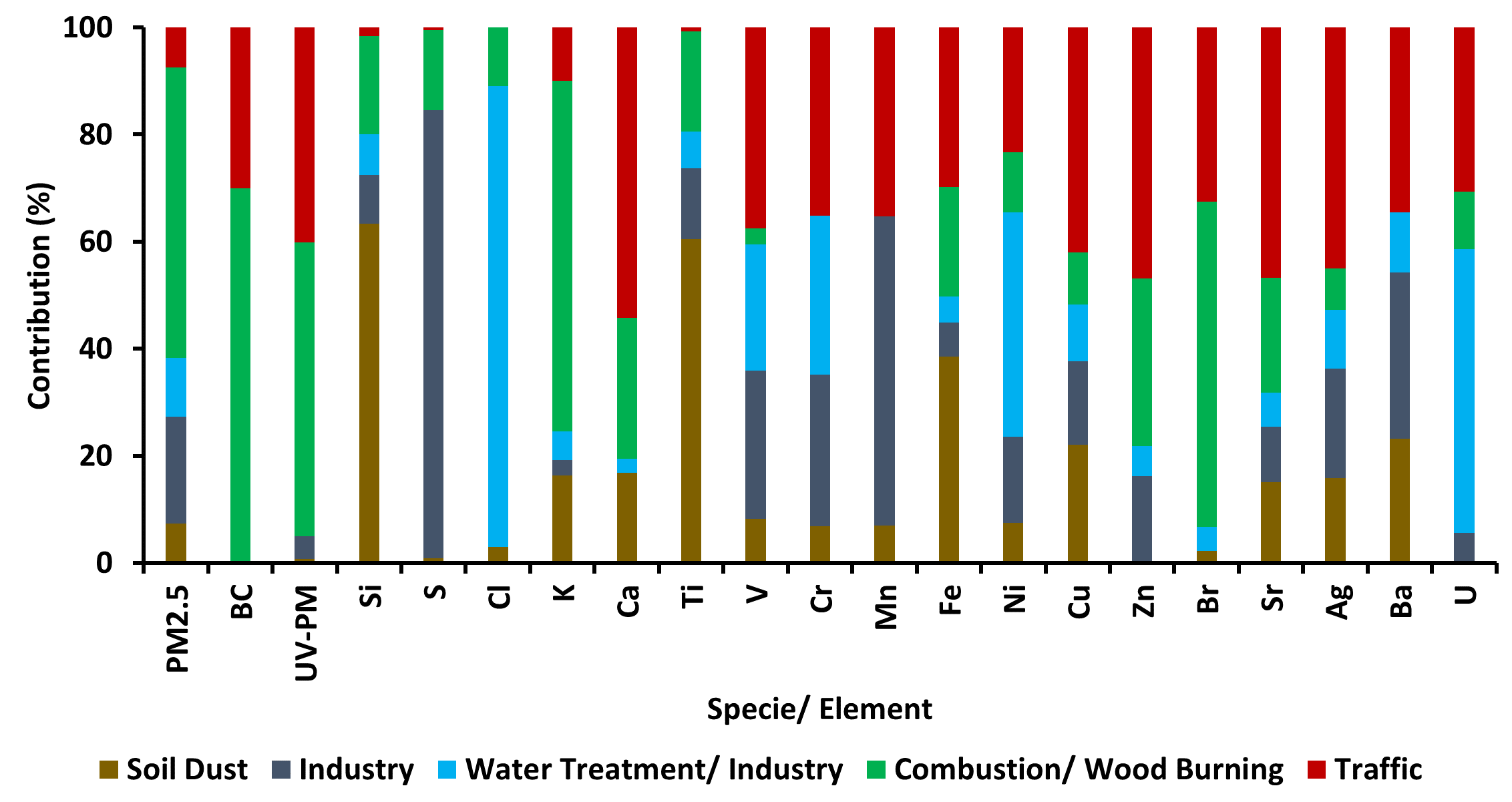


Figure S2 Source profile for each factor, showing percentage contributions of the species. The five factors are explained in the text.


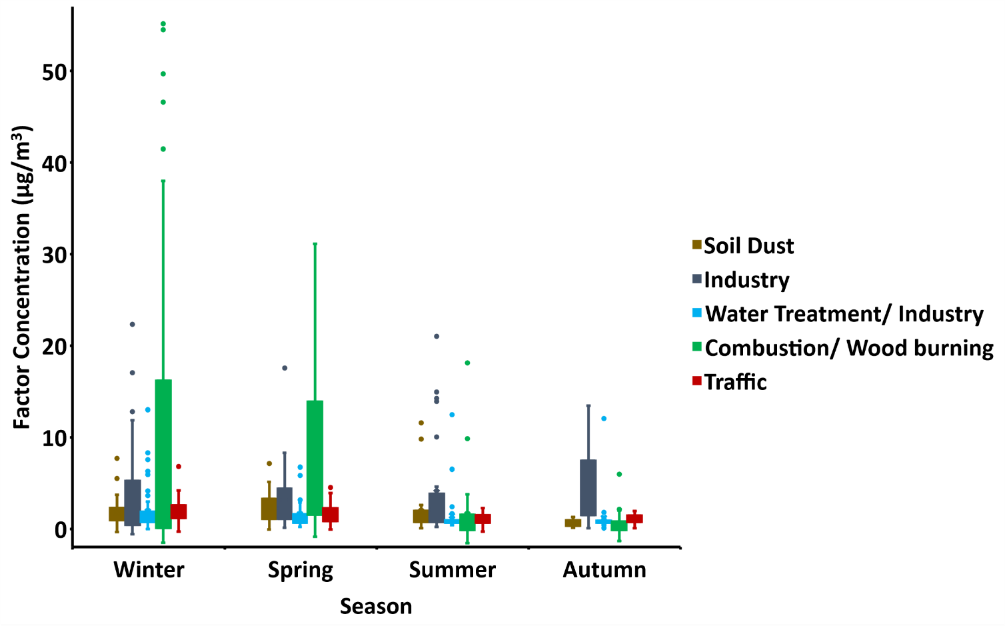


Figure S3 Seasonality of each factor, by means of data variation.


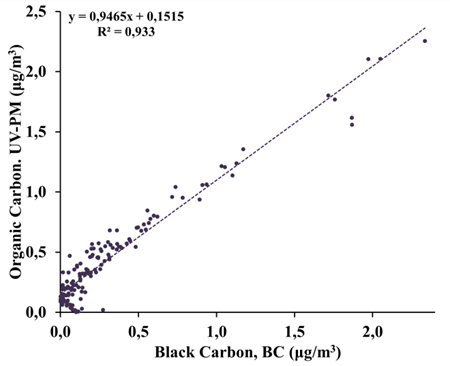


**Figure S4** Correlation between Organic Carbon (UV-PM) and Black Carbon concentrations from June-16 2020 to August 2-18 2021 in Bloemfontein, South Africa.


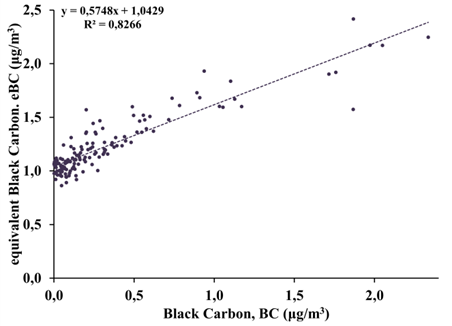


**Figure S5** Correlation between soot and Black Carbon concentrations from June-16 2020 to August-18 2021 in Bloemfontein, South Africa.


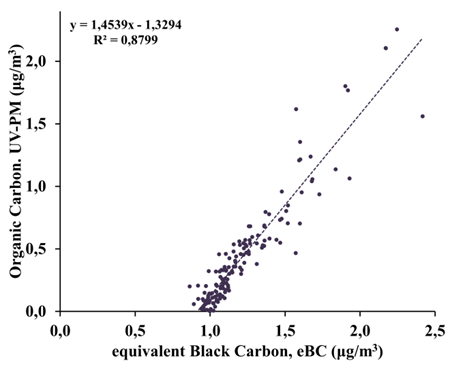


**Figure S6** Correlation between Organic Carbon (UV-PM) and soot concentrations from June-16 2020 to August-18 2021 in Bloemfontein, South Africa.

**Table S1** Spearman correlations between BC, UV-PM, elements and PM_2.5_* statistically significant p<0.05.

|  | PM_2.5_ | BC | UV-PM |
| --- | --- | --- | --- |
|  |  |  |  |
| PM_2.5_ | 1 |  |  |
| BC | 0.4257* | 1 |  |
| UV-PM | 0.5087* | 0.9540* | 1 |
| Ag | -0.0622 | -0.0484 | -0.2346* |
| Ba | -0.0857 | 0.1823* | -0.1646* |
| Br | 0.4906* | 0.8161* | -0.0622 |
| Ca | 0.3480* | 0.5829* | -0.0857 |
| Cl | 0.1616 | 0.4685* | 0.4906* |
| Cr | -0.2326* | -0.1831* | 0.3480* |
| Cu | -0.0417 | 0.2009* | 0.1616 |
| Fe | 0.3572* | 0.6406* | -0.2326* |
| K | 0.5076* | 0.6891* | -0.0417 |
| Mn | -0.4319* | -0.3651* | 0.3572* |
| Ni | 0.2710* | 0.0556 | 0.5076* |
| P | 0.161 | 0.2978* | -0.4319* |
| S | 0.3352* | 0.2635* | 0.2710* |
| Si | 0.3733* | 0.5216* | 0.161 |
| Sr | 0.3186* | 0.1491 | 0.3352* |
| Ti | 0.3406* | 0.4392* | 0.3733* |
| U | -0.0784 | 0.1355 | 0.3186* |
| V | -0.144 | -0.1667* | 0.3406* |
| Zn | 0.3416* | 0.6492* | 0.3416* |


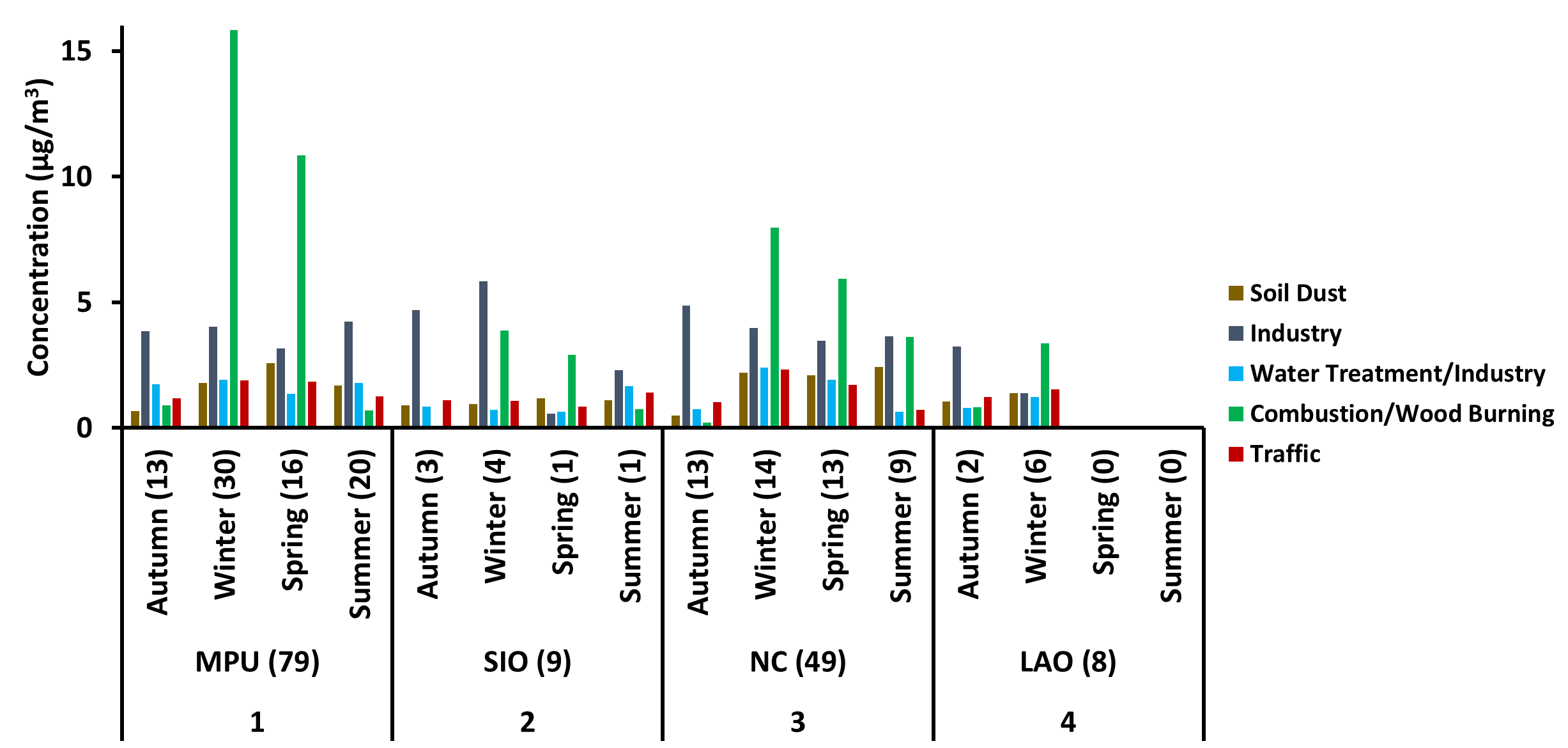


Figure S7 Seasonality of the MP, SIO, NC an LAO clusters, and factorial contribution to the cluster.
